# Beef-Tea: Its Body and Soul

**Published:** 1895-07-27

**Authors:** 


### Beef-Tea: Its Body and Soul.

Beef-tea is so much a matter of course in invalid diet that it is a matter of some importance that it should be as well made as possible; that is, that it should contain a maximum amount of nutriment in the most palatable form. Some of us remember the greasy liquid, faintly tinged with yellow, and with a little flocculent matter reposing at the bottom of the basin, which is occasionally offered to the invalid as beef-tea by a self-satisfied landlady. And yet, to read that landlady's account for "tea-beef" one would think that the concentrated strength of a whole ox was in every meal. The good woman probably is not dishonest, but she has never learned that beef-tea is something more than the liquor in which beef has been boiled. Nevertheless it is not fair to condemn her for not producing the beef-tea of the laboratory, a liquid for which her appliances are as scant as her knowledge. After all the invalid might not be much better off if he fell into the hands of the chemist instead of the cook. The chemist may probably succeed in putting more albumen into the tea, and it is the albumen which contains the nutriment desired—the body, as we have chosen to name it—but he may have omitted from his solution those salts and extractives which make it palatable, and which we have styled its soul. To combine the two in the highest possible degree is the aim of the invalid's cook.

By a process of cold extraction the largest amount of albumen can be obtained, and it has, therefore, been recommended on the ground of economy, as particularly suitable for hospital use. A committee of investigation which studied the Newcastle Infirmary in 1886 thought that ten ounces of meat to the pint of tea would be sufficient "if it be cooked at a lower temperature, and for a longer time." The analyst of the city of Carlisle when appealed to declared that he could, by a process of his own, obtain about six times the amount of albumen in the tea from the same quantity of beef. "This deficiency of albumen is of importance," he said, "as the whole of the nutritive value of the tea depends on the albumen, the extract, consisting of flavouring matters, salts, and gelatine, experiment has proved (what we should expect) that it is only a stimulant, and has little or no nutritive value."

That stimulants are of use in illness is not to be denied, and it may often be desirable to give stimulants which are not intoxicants, but beef-tea is supposed to be a food, and as such it is given. But the stimulant parts of the beef—the extractives and salts—are valuable as containing the palatable quality which induces an invalid to take the whole, albumen alone being tasteless, however nutritive. To obtain the combination, it sometimes seems that some albumen must be lost; for though by treating the beef with a few drops of hydrochloric acid in the water, more albumen can be extracted, the result is deficient in proportionate salts necessary to make the tea savoury. Moreover, the average housekeeper has a healthy dread of mixing drugs with her cookery, and even if beef-tea made with hydrochloric acid be possible in the laboratory, it will never be popular in the kitchen.

Some experiments made by Dr. Phillips Bedson, Professor of Chemistry in the Durham College of Science at Newcastle-on-Tyne, give the results of the various methods, scientific and homely, of making beef-tea. First he made it in the general way, allowing very nearly a pound to a pint—to be accurate, 19 lbs. of beef to twenty pints of water. The beef was allowed to soak in the water for some hours, then both together were gradually warmed to near boiling point. By this he obtained 43·7 grains of albumen to each pint, 78·29 grains of gelatine and extractives insoluble in alcohol, 75·7 grains of soluble extractives, and 31·6 grains of salts. A large proportion of fat in his first extraction (147·9 grains) should not be reckoned in the result, as by rights this would be removed before the tea was used. A second infusion made the same way, but with the fat removed, gave only 1·73 grains of fat.

Dr. Bedson next tried a method of cold infusion, in the proportion of 10 ozs. of meat to one pint of water. This proportion of meat had been recommended to the managers of the Newcastle Infirmary, and for the sake of economy they were willing to adopt it if the result was satisfactory. The extraction was made in the cold, and the cold extract poured off from the meat before it was cooked. "In this way," says Dr. Bedson, "a tea was obtained of a brownish colour, tasteless and insipid, and holding in suspension a light flocculent precipitate of coagulated albumen." This hardly sounds like a dish to tempt an invalid's appetite; and, when analysed, it proved to be deficient in some of the requisites of good beef-tea. The result ran thus:—

| | | |
|---|---|---|
| Albumen ... ... ... ... ... | 39·3 | grains per pint |
| Gelatine, &c. (insoluble in alcohol) | 21·3 | ,, ,, |
| Extractives (soluble in alcohol) ... | 64·4 | ,, ,, |
| Fat ... ... ... ... ... ... | 3·5 | ,, ,, |
| Salts ... ... ... ... ... ... | 6·6 | ,, ,, |

The smaller quantity of meat allowed per pint would partly account for the poverty of the extract, but the method of extraction is also to blame. Another cold extraction also proved unsatisfactory.

Dr. Bedson tried yet another method, which seems to combine, as far as possible, the advantages of both the cold and hot methods of extraction. It is of the stronger make, a pound of beef to make a pint of tea, below which it is probable that beef-tea cannot be satisfactory. By this method a pound of finely minced beef was first mixed with half a pint of cold water and left for twelve hours. The liquid was then strained off and cooked, and a second half-pint of water added. This was allowed to remain with the meat for three hours, and then the whole, meat as well as liquor, gradually heated to near the boiling-point. This tea was then strained off, and added to the first with sufficient water to allow for any loss by evaporation. The analysis of this showed—

| | | |
|---|---|---|
| Albumen ... ... ... ... ... | 67 8 | grains per pint |
| Gelatine, etc. (insoluble in alcohol) | 33·78 | ,, ,, |
| Extractives (soluble in alcohol) ... | 100·9 | ,, ,, |
| Fat ... ... ... ... ... ... | 56·3 | ,, ,, |
| Salts ... ... ... ... ... ... | 35·08 | ,, ,, |

altogether 293·86 grains of solids for each pint of tea. Moreover, this tea is said to be palatable and agreeable; and there is nothing abnormally difficult in the method of making. It takes a long time, it is true, but good beef-tea cannot be made in a hurry. In an emergency the first extraction might be used alone, and the second added to the first drawing of a new "brew." Thus the full strength could be secured and waste avoided. But this need apply only to private families. In hospitals, where the making of beef-tea is an every-day duty, there should be no difficulty in producing a tea which should give the highest nutritive value attainable in a palatable form.

